# FGF21–MAPK1 Imbalance Disrupts Hepatic Lipid Metabolism in Dairy Cow Ketosis

**DOI:** 10.3390/life15091339

**Published:** 2025-08-24

**Authors:** Jun-Jie Xu, Fan Yang, Zhi-Xi Chen, Zhi-Peng Wang, Zi-Xuan Wang, Zi-Han Deng, Chen-Jie Xu, Fang-Hui Chen, Wei Zhang, Yang Liu, Ya-Fei Cai

**Affiliations:** 1 College of Animal Science and Technology, Nanjing Agricultural University, Nanjing 210095, China; 2023205002@stu.njau.edu.cn (J.-J.X.); chenzhxii@163.com (Z.-X.C.); 2023105002@stu.njau.edu.cn (Z.-P.W.); 2023205001@stu.njau.edu.cn (Z.-X.W.); 18186965525@163.com (Z.-H.D.); 2019105001@njau.edu.cn (C.-J.X.); fchen31@emory.edu (F.-H.C.); weizhang@njau.edu.cn (W.Z.); yangliu@njau.edu.cn (Y.L.); 2 Department of Human Anatomy, Bengbu Medical University, Bengbu 233030, China; bridgeyangfan11111@126.com

**Keywords:** dairy cows, ketosis, lipid metabolism, MAPK1, FGF21

## Abstract

Background: Aberrant hepatic lipid metabolism is a key predisposing factor for dairy cow ketosis, with genetic factors playing a pivotal role in disease pathogenesis. However, systematic screening and functional validation of candidate genes for bovine ketosis remain limited. In this study, we aimed to identify genetic markers associated with clinical ketosis and explore their potential functional mechanisms underlying disease susceptibility. Methods: We conducted simplified genome sequencing (SuperGBS), genome-wide association studies (GWAS), and Sanger sequencing on Chinese Holstein cows, both healthy and with ketosis. Results: We reported that mitogen-activated protein kinase 1 (MAPK1) was significantly associated with clinical ketosis. Further investigation revealed concurrent upregulation of MAPK1 protein and disrupted hepatic lipid homeostasis in hepatocytes from in vivo and in vitro models. Critically, siRNA-mediated knockdown of MAPK1 reversed lipid metabolism processes and reduced lipid accumulation in β-Hydroxybutyric acid (BHB)-exposed bovine hepatocytes, thereby establishing MAPK1 activation as a driver of lipotoxicity in dairy cow ketosis. Additionally, we identified that supplementation of fibroblast growth factor 21 (FGF21) fusion protein not only reduced MAPK1 expression but also normalized hepatic lipid metabolism in BHB-exposed bovine hepatocytes. Conclusions: FGF21–MAPK1 imbalance is a reason for hepatic lipid metabolic dysfunction, providing a potential intervention approach to mitigate dairy cows’ ketosis.

## 1. Introduction

Ketosis is a prevalent metabolic disorder in dairy cows, with a global prevalence approximating 20% [1,2]. It causes significant reductions in milk yield and reproductive efficiency [3,4], and it triggers secondary diseases like abomasal displacement and mastitis, leading to significant economic losses in the dairy sector [5,6]. The diagnostic gold standard for ketosis remains blood β-Hydroxybutyric acid (BHB) concentration (≥1.2 mmol/L for clinical cases) [7]. Notably, milk BHB demonstrates strong correlation with blood levels (R^2^ = 0.5419), enabling non-invasive screening at a practical cutoff of ≥0.2 mmol/L [8]. Beyond diagnosis, emerging evidence reveals that exogenous BHB administration (2–4 mM in vitro) can induce a ketosis-like metabolic state in bovine hepatocytes [9]. This experimental paradigm has been instrumental in elucidating ketosis pathogenesis. Thus, BHB serves not only as biomarker for early detection but also as an experimental inducer to dissect ketosis pathophysiology in dairy cows.

Substantial evidence indicates that hepatic lipid metabolism dysfunction is the hallmark of bovine ketosis [10]. In ketotic cows, previous studies revealed a coordinated upregulation of lipogenic regulators, such as fatty acid synthase (FASN) (2–3 fold) and Stearoyl-CoA desaturase-1 (SCD1) (2–3 fold), alongside downregulation of fatty acid oxidation regulators, exemplified by Carnitine palmitoyl transferase 1a (CPT1a) (3–4 fold) in liver tissues [9,11,12]. This imbalance manifests as impaired hepatic β-oxidation capacity and intracellular triglycerides accumulation, which directly correlates with blood BHB concentrations.

Emerging research implicates mitogen-activated protein kinase 1 (MAPK1) as a potential regulatory factor of intracellular lipid metabolic homeostasis [13]. Previous studies have shown that MAPK1 increases the formation of lipid droplets in adipocytes by phosphorylating dynein, which increased the amount of the protein in adipose differentiation-related protein (ADRP)-containing lipid droplets [14]. It reflects that in lung cancer cell lines, the activation of MAPK1 (e.g., via arachidonic acid treatment) reduces lipid droplet formation through inhibiting the expression of FASN and proliferator-activated receptor gamma (PPARγ) [15]. These regulatory roles converge to produce a significant functional effect in lipid handling. Notably, multiple studies have reported that MAPK1 serves as a key mediator of fibroblast growth factor 21 (FGF21)-dependent lipid metabolism regulation. FGF21, a pleiotropic metabolic regulator identified as a putative biomarker for dairy cow ketosis [16], exerts its beneficial effects by enhancing hepatic fatty acid oxidation and improving peripheral insulin sensitivity [17]. Mechanistically, MAPK1 interacts with FGF21 through phosphorylation-dependent signaling cascades, thereby modulating downstream targets involved in lipid homeostasis and energy balance [18,19]. However, the precise involvement of FGF21–MAPK1-mediated hepatic lipid homeostasis in ketosis development warrants clarification.

In the present study, we employed an integrative approach, combining SuperGBS sequencing, GWAS, and Sanger sequencing to identify MAPK1 as a candidate gene associated with dairy cow ketosis through comprehensive sequencing and bioinformatics analyses. Furthermore, using a BHB-induced in vitro model, we demonstrated that reversing FGF21–MAPK1 pathway dysregulation effectively restores hepatic lipid homeostasis. These findings suggest that FGF21 fusion proteins targeting the FGF21–MAPK1 axis represent promising therapeutic candidates for managing ketosis in dairy cows.

## 2. Materials and Methods

### 2.1. Sequencing Sample Acquisition and Initial Handling

The blood samples used for sequencing were obtained from Chinese Holstein adult cows that had calved two or more times at a dairy farm in Jiangsu Province, China, with ages ranging from 2.5 to 5 years (the selection criteria for experimental animals in the following experiments were consistent with those here). Relying on the Dairy Herd Improvement (DHI) Center of Jiangsu Province, we tracked the production performance measurement indicators of the dairy cows in this pasture within 30 days after each calving from 2017 to 2019. We selected two groups of cows: healthy cattle (those with postpartum BHB levels < 0.15 mmol/L for three consecutive calvings) and cattle with clinical ketosis (those with postpartum BHB levels ≥ 0.2 mmol/L for 3 consecutive calving). Coccygeal vein blood samples were uniformly collected from both groups of cows (*n* = 20 from healthy cows and *n* = 25 from cows with clinical ketosis).

After DNA extraction (with an OD_260_/OD_280_ ratio of 1.8–2.0), the samples were tested for quality using agarose gel electrophoresis, and the quality results were analyzed (Supplementary Table S1). The results of the agarose gel electrophoresis test showed that all 45 DNA samples had neat bands with no trailing phenomenon, and their quality was classified as class A, confirming their suitability for sequencing. Subsequently, we processed the Raw Reads by splitting them into individual samples. We used the fastx_trimmer program (from the FastX Toolkit) to remove restriction enzyme cleavage sites and 3′-end bases with FastQC quality scores < 20, thereby obtaining quality-controlled Clean Reads.

### 2.2. SNP Typing and Bioinformatics Analysis Based on SuperGBS Sequencing

After the sample DNA extraction is qualified, the SuperGBS sequencing library is constructed according to the following steps: (1) Enzymatic digestion: Restriction endonucleases PstI-HF/MspI are used for the digestion process. (2) Ligation: We added adapters and barcodes for the digestion process to both ends of the DNA fragments using T4 ligase. (3) Recovery: An improved magnetic bead recovery system is employed to recover the fragments. (4) Amplification: A high-fidelity enzyme is utilized for PCR amplification of the recovered fragments. (5) Detection: The determination of the PCR product is determined, and it must be greater than 5 ng/μL. (6) Test delivery: The library is mixed and then subjected to on-board sequencing using the Illumina Hiseq Xten sequencing system (Illumina, Inc., San Diego, CA, USA) with paired-end 150 (PE150) reads.

After sequencing, we obtained Raw Reads. Following quality control of these Raw Reads, we obtained Clean Reads, which were then aligned to the reference genome (*Bos taurus* UMD 3.1.1) using Bowtie2 (version 2.5.4). Based on the alignment results, we used GATK (version 4.5.0.0) to detect and screen single-nucleotide polymorphisms (SNPs), thereby obtaining initial SNPs calls. The SNPs were genetically annotated using SnpEff (version 4.3t). Principal component analysis (PCA) was performed using PLINK2 (version 2.00a2.3) to obtain eigenvectors. Population structure analysis was performed for K values ranging from 2 to 10 using ADMIXTURE (version 1.3.0). Then, VCFtools (version 0.1.16) was used to calculate the *p*-value of Harwen equilibrium. A *p* > 0.05 indicates that the investigated population has reached genetic balance. Subsequently, the population diversity index such as nucleotide diversity (Pi) and the genetic differentiation coefficient (Fst) among populations were calculated. Differential SNP clusters were plotted using the heatmap package in R. For linkage disequilibrium analysis, PopLDdecay (version 3.40) software (with the parameter MaxDist set to 1000 and other parameters set to defaults) was used to calculate the different spacing r^2^ values of each subgroup. Then, the Plot_MultiPop.pl script in PopLDdecay (with the parameter bin2 set to 1000 and other parameters set to defaults) was used to generate the subgroup r^2^ value for each subgroup after subgroup binning. The fit was plotted using the ggplot2 (version 3.5.1) package in R. SuperGBS was completed by Oebiotech (Shanghai, China).

### 2.3. Genome-Wide Association Analysis of SNPs with Clinical Ketosis

The BHB concentration in milk, which was measured for the first time within 30 days postpartum in the two groups of cows in 2019, was used as the dependent variable for GWAS. EMMAX (version beta-07Mar2010) was employed to apply a mixed linear model. A quantile–quantile (QQ) plot was generated to evaluate the association analysis. Additionally, Manhattan plots were utilized to present the results of the association analysis.

### 2.4. Independent Sample Study

According to the same ketosis criteria, blood samples were collected from dairy cows in different pastures across Jiangsu Province. The sample group consisted of 48 cattle with clinical ketosis and 136 healthy cattle. DNA was then extracted from these sample with the values OD_260_/OD_280_ = 1.7–2.0. To identify SNP markers related to clinical ketosis, relevant primers were designed using the primer designing tool on the NCBI website. Subsequently, PCR amplification was performed. After that, Applied Biosystems 3730xl DNA Analyzer (Azenta, Inc., Burlington, MA, USA) was en-trusted to perform direct sequencing. Chromas 2.6.6 software was used to verify the SNP genotyping results. Based on the genotyping results, a case-control study was conducted to further identify SNP markers associated with clinical ketosis in dairy cows. The PCR reactions were performed in a volume of 30 μL, which included 15 μL of 2×Rapid Taq Master Mix (#P222, Vazyme Biotech Co., Ltd., Nanjing, Jiangsu, China), 1.2 μL each of the forward and reverse primer, 100 ng of template DNA, and ddH_2_O to make up the total volume to 30 μL. The PCR reaction program was as follows: an initial pre-denaturation step at 95 °C for 5 min; followed by 30 cycles, each consisting of denaturation at 95 °C for 15 s, annealing at the specified annealing temperature for 15 s, and extension at 72 °C for 7 s; and a final extension step at 72 °C for 5 min. The primers and their corresponding annealing temperatures are provided in Supplemental Table S2. Sanger sequencing was completed by Sangon Biotech (Shanghai, China) Co., Ltd.

### 2.5. Tissues and Cell

The liver tissues of cows (healthy: *n* = 3; ketosis: *n* = 3) were collected from Nanjing Linling Meat Industry Co., Ltd., Jiangning District, Nanjing City, Jiangsu Province, China. The animal testing protocol for this study was approved by the Animal Welfare and Ethics Committee of Nanjing Agricultural University (SYXK-2017-0027). Bovine hepatocytes were purchased from YaJi Biological, Shanghai, China. These bovine hepatocytes were cultured in Dulbecco’s minimum essential medium supplemented with 10% fetal bovine serum and 1% penicillin–streptomycin solution. The culture was then incubated at 37 °C in a humidified atmosphere containing 5% CO_2_, and the medium was replaced every 48 h.

### 2.6. Oil Red O Staining for Visualizing Lipid Morphology

The lipid morphology of both liver tissues and bovine hepatocytes stimulated with BHB was visualized by Oil Red O staining. First, fresh liver tissues were rapidly fixed in 4% paraformaldehyde overnight at room temperature and embedded in paraffin. Subsequently, the liver tissues were sectioned into 4 μm thick slices, which were processed for Oil Red O (Sigma) staining. Both the tissue sections and cell monolayers after BHB stimulation were stained with Oil Red O, in accordance with the manufacturer’s instructions.

### 2.7. Quantitative Real-Time Polymerase Chain Reaction (qPCR) Analysis

Total RNA was extracted from liver tissue and bovine hepatocytes using TRIzol reagent (Invitrogen, Carlsbad, CA, USA), following the manufacturer’s instructions. Subsequently, the extracted total RNA was reverse-transcribed into cDNA according to the protocol of the First-Strand Synthesis System (PrimeScriptTM RT Master Mix, TaKaRa, Kusatsu, Shiga, Japan). The qRT-PCR amplification was carried out using the QuantStudio 7 Real-Time PCR System (Applied Biosystems, Foster City, CA, USA) with the specified primers (Supplemental Table S3). The relative transcript abundance was determined using the 2^−ΔΔCt^ method, and the results were normalized using the housekeeping gene *β-Actin* as a reference.

### 2.8. Western Blotting (WB) Analysis

The traditional RIPA lysis buffer method (including PMSF and cocktail) was used to extract the total protein from liver tissue and bovine hepatocytes (Cat#P0013C, Beyotime Biotechnology, Shanghai, China). The total protein concentration was then determined using the BCA protein assay kit (Cat#P0012, Beyotime Biotechnology, Shanghai, China). The antibodies used in this study were as follows: Anti-MAPK1 (Cat#D264559, Sangon Biotech (Shanghai, China) Co., Ltd., Shanghai, China), Anti-FASN (Cat#66591-1-Ig, Proteintect, Wuhan, China), Anti-CPT1a (Cat#15184-1-AP, Proteintect, Wuhan, China), Anti-ACOX1 (Cat#D121471, Sangon Biotech (Shanghai, China) Co., Ltd., Shanghai, China), Anti-SCD1 (Cat#D162163, Sangon Biotech (Shanghai, China) Co., Ltd., Shanghai, China), Anti-FGF21 (Cat#A3908, ABclonal, Wuhan, China), Anti-α-Tubulin (Cat#2125S, Cell Signaling Technology, Danvers, MA, USA), HRP-conjugated Goat Anti-Rabbit IgG (Cat#D110058, Sangon Biotech (Shanghai, China) Co., Ltd., Shanghai, China), and HRP-conjugated Affinipure Goat Anti-Mouse IgG(H+L) (Cat#SA00001-1, Proteintech, Wuhan, China).

The proteins were boiled in SDS-PAGE sample buffer (62.5 mM Tris-HCl, pH 6.8; 2% SDS; 10% glycerol; and 0.1% bromophenol blue) supplemented with 5% β-mercaptoethanol (β-ME). Subsequently, they were separated by SDS-PAGE and transferred onto PVDF membranes (Bio-Rad, Hercules, CA, USA). The membranes were blocked with 5% non-fat milk in PBS for 1 h at room temperature. After that, they were incubated with primary antibodies at 4 °C overnight, followed by incubation with the appropriate HRP-conjugated secondary antibodies for 1 h at room temperature. Finally, the membranes were washed and subjected to detection.

### 2.9. Bovine Hepatocytes Exposed with BHB

After being cultured until they reached 80% confluence, the cells were subjected to serum starvation for 12 h. Subsequently, the bovine hepatocytes were exposed to BHB at concentrations of 0, 0.6, 1.2, or 2.4 mmol/L. The exposure to 1.2 mmol/L and 2.4 mmol/L represents subclinical and clinical ketosis, respectively. Following 12 h BHB exposure, total RNA and protein were extracted from the cells. These extracts were then used for the quantification of mRNA levels (by qPCR) and protein expression (by Western blot) of target metabolic genes.

### 2.10. RNA Interference

Since the single-nucleotide polymorphism MAPK1 (rs74014223, T > G) ranked the highest in the GWAS, bovine hepatocytes were transfected with MAPK1 small interfering RNA (siRNA) to study the role of this gene in lipid metabolism. Three pairs of siRNA sequences were designed to induce post-transcriptional silencing of the MAPK1 gene in bovine hepatocytes (Supplemental Table S4). These MAPK1 siRNA sequences were then transfected into bovine hepatocytes using Lipofectamine^TM^ 3000 Reagent (Cat#L3000015, Invitrogen, Carlsbad, CA, USA). The specific operational steps followed the instructions provided with the transfection reagent. The cell concentration used for the transfection experiments was 5 × 10^5^ cells/mL. To study the effect of MAPK1 on lipid metabolism genes in bovine hepatocytes, four different treatment groups were designed in this experiment: the negative control group (siNC), the MAPK1 siRNA group (siRNA), the NC + BHB group (siNC + BHB), and the MAPK1 siRNA + BHB group (siRNA + BHB). According to a previous study, MAPK1 mRNA and protein expression increased most significantly when the final BHB concentration was 2.4 mmol/L. Therefore, in this study, after 36 h of siRNA treatment, the siNC + BHB group and the siRNA + BHB group were treated with BHB for 12 h (with a final BHB concentration of 2.4 mmol/L). After a total of 48 h, the cells were collected, total protein was extracted, and protein expression was detected.

### 2.11. FGF21-Fc Fusion Protein Treatment

After bovine hepatocytes were exposed with 2.4 mmol/L BHB and cultured for an additional 12 h, they were treated with FGF21-Fc fusion protein at concentrations of 0, 1.25, 2.5, 5, 10, and 20 mg/L. Following another 12 h culture period, total protein was collected to detect the expression levels of FGF21, MAPK1, FASN, SCD1, CPT1a, and ACOX1.

### 2.12. Statistical Analysis

A case-control study was conducted, and the chi-square test was applied using SPSS21.0 software. An independent sample *t*-test was used to detect differences in mRNA and protein expression. The relative expression levels were plotted using GraphPad Prism 8.0. For the Western blotting (WB) images, ImageJ software was utilized. Based on the intensity of the target bands in the WB images, density values were converted into specific numerical values to facilitate the comparison of protein-level differences. All data were analyzed using Student’s *t*-test and one-way analysis of variance (ANOVA). * *p* < 0.05, ** *p* < 0.01, *** *p* < 0.001. Lowercase letters indicate significant level (*p* = 0.05). Differences are considered not significant if there is one letter with the same label, and significant if there are different letters with different labels.

## 3. Results

### 3.1. Association Analysis Between Seventeen SNPs and Glucose and Lipid Metabolism Genes in Chinese Holstein Cows

**Figure 1:**
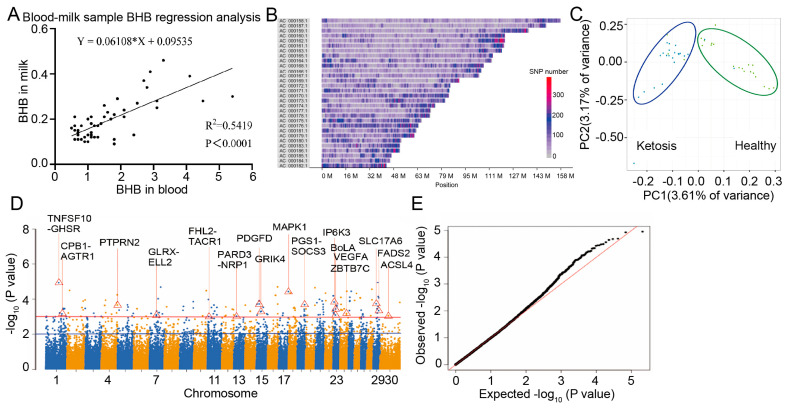
SuperGBS sequencing and GWAS analysis of Chinese Holstein cows. (**A**) Linear regression analysis of BHB in blood and milk. (**B**) Distribution of SNPs on chromosomes: ordinate is sequence number, abscissa is sequence physical location, and different colors represent the number of SNPS in this window. (**C**) Principal component analysis of SNPs: the horizontal and vertical coordinates represent two principal components, each point is a sample, and different shapes and colors represent different groups. (**D**) Manhattan map of SNP effect in mixed linear model and 17 candidate genes associated with glucolipid metabolism and insulin resistance: the horizontal coordinate is the genomic coordinate, and the vertical coordinate is the negative logarithm (−log10p) of the associated *p*-value of each SNP. (**E**) Quantile–quantile plot: the horizontal coordinate is the expected value, and the vertical coordinate is the observed value. The black scatter was attached to the red solid line at the front and only warped at the end, representing a low SNP false positive rate, and the correlation analysis results are relatively reliable.

In the present study, we aimed to investigate the genetic factors associated with clinical ketosis in Chinese Holstein cows. To begin with, 57 lactating dairy cows were randomly selected from the farm, Blood and milk samples were collected from these cows, and the levels of blood BHB and milk BHB levels were determined separately. To assess the feasibility of using milk BHB levels as an indicator for clinical ketosis diagnosis, linear regression analysis was conducted to evaluate their relationship. The results revealed a highly significant linear correlation between blood and milk BHB (*p* < 0.0001), with an R-square of 0.5419 (Figure 1A). This finding proved a solid basis for using milk BHB levels to determine whether Chinese Holstein cows were suffering from clinical ketosis. According to the diagnostic criteria specified in the industry standard (NYT 3191-2018, http://www.najs.org, accessed on 23 July 2025), which states that cows with clinical ketosis have a blood BHB level of ≥1.6 mmol/L, the milk sample threshold for diagnosing clinical ketosis in dairy cows in the present study was set at milk BHB > 0.19 mmol/L. This threshold was consistent with the findings of previous studies [8,20].

Based on the milk BHB levels as the grouping criterion, the cows were divided into a ketosis group (25 cows) and a healthy group (20 cows). Blood samples were then collected from the tail veins of these cows. Subsequently, SuperGBS sequencing on these samples was performed. Bioinformatics (version 2.0) was employed to screen, filter, and perform quality control on the genotyping results, ultimately obtaining 360,666 SNPs. Figure 1B illustrates the distribution of these SNPs across the chromosomes. To explore the genetic differences between the ketosis and healthy groups, principal component analysis (PCA) based on the SNP differences among samples was performed (Figure 1C). The results showed that the two largest eigenvectors separated the samples into two distinct subgroups, which clearly corresponded to the healthy and ketosis groups. This indicated that there were significant genetic differences between the two groups, providing a foundation for further genetic analysis.

After obtaining a sufficient number of SNPs, GWAS were performed using 103,002 filtered SNPs to explore their correlation with clinical ketosis in Chinese Holstein cows. The phenotype used for this analysis was the blood BHB concentration in milk samples collected within 30 days postpartum, as ketosis in dairy cows is closely associated with glycolipid metabolism during this period. We conducted a functional screening of genes annotated in the GWAS analysis at a significance level of *p* < 0.001. As a result, we finally identified 17 genes related to ketosis in Chinese Holstein cows. As shown in Table 1, these candidate genes were primarily associated with insulin/glucose/lipid metabolism and the pathogenesis of diabetes, which are all closely related to the development of ketosis. Among 17 SNPs, 16 were localized on autosomes, and 1 was on the X chromosome. After the preliminary identification of the candidate genes, they were labeled on the Manhattan plot of GWAS according to the distribution of the SNPs (Figure 1D). The full characteristics of the SNPs are detailed in Table 2. Figure 1D shows the results of the GWAS and the 17 candidate genes associated with glucose-lipid metabolism and insulin resistance. The number of SNPs with *p* < 0.01 is above the blue solid line, totaling 1186, and the number of SNPs with *p* < 0.001 is above the red solid line, totaling 177. To evaluate the reliability of the GWAS results, we examined the quantile–quantile (QQ) plot (Figure 1E). The results showed that the scatter distribution was only warped at the end of the red solid line, indicating that the rate of false positive SNPs was low. This demonstrated that the mixed linear model used for the association analysis was highly feasible. In summary, we successfully screened 17 candidate genes for ketosis in Chinese Holstein cows.

### 3.2. Association Analysis Between Four Candidate Genes and Clinical Ketosis in Chinese Holstein Cows

**Figure 2:**
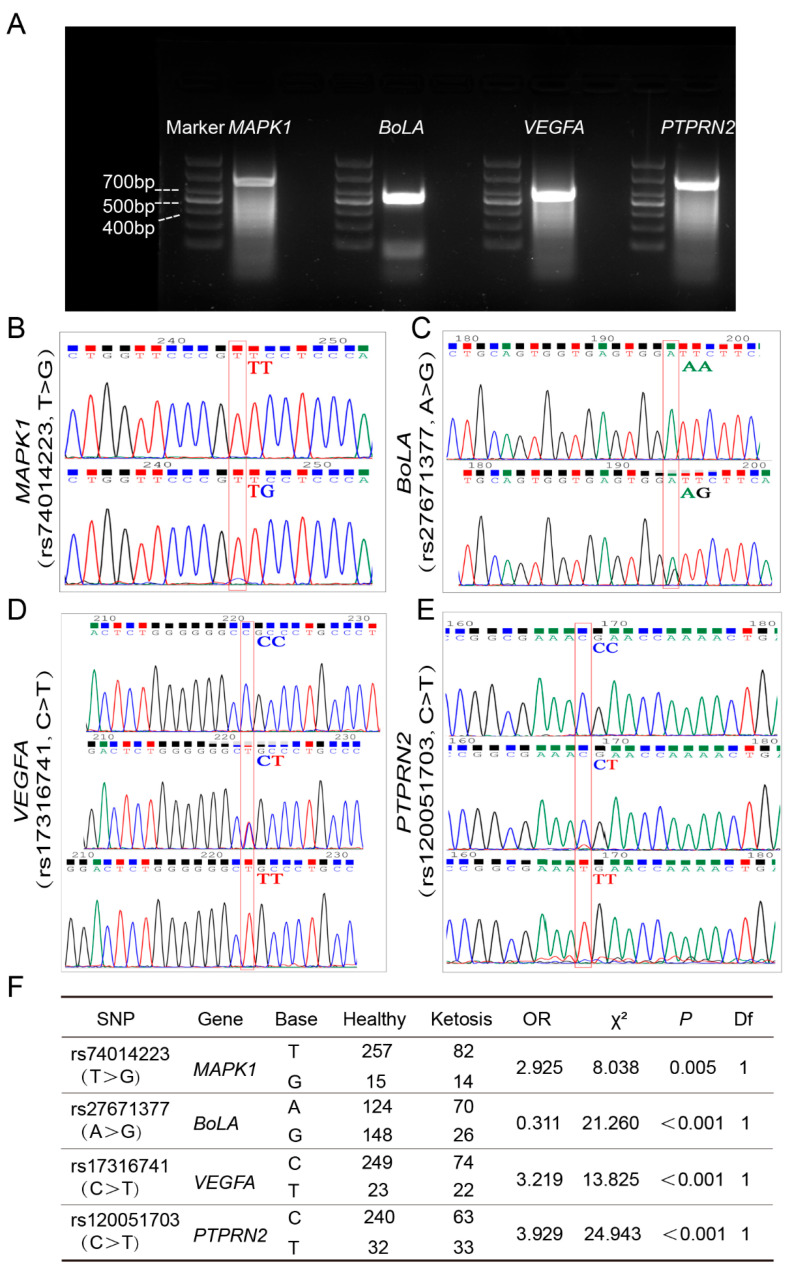
Four SNPs showed significant differences in Sanger sequencing of Chinese Holstein cows. (**A**) Gel electrophoresis pattern of PCR-amplified fragments near four significant SNPs. There were PCR-amplified fragments of the SNPs *MAPK* (rs74014223), *BoLA* (rs27671377), *VEGFA* (rs17316741), and *PTPRN2* (rs120051703) regions, respectively. M: DL1000: DNA marker. (**B**) PCR amplification fragment containing *MAPK1* (rs74014223, T > G). The typing results were TT and TG. (**C**) PCR amplification fragment containing *BoLA* (rs27671377, A > G), genotype AA and AG. (**D**) PCR amplification fragment containing *VEGFA* (rs17316741, C > T) with CC, CT, and TT typing. (**E**) PCR-amplified fragment containing *PTPRN2* (rs120051703, C > T). The typing results were CC, CT, and TT. (**F**) Four SNPs showed significant differences in Sanger sequencing of Chinese Holstein cows (*p* < 0.05; healthy control *n* = 136, ketosis *n* = 48).

To further refine this set of SNP markers and enhance confidence in their association with ketosis, the following experiments were adopted. First, Sanger sequencing was performed on an independent sample group, providing an initial validation of the previously identified SNPs in a new set of samples, and reducing the likelihood of false positive associations. Subsequently, a case-control study was designed. Whole blood samples were collected from dairy cows on farms in Jiangsu Province, including 48 cows with clinical ketosis and 136 healthy cows, using the same diagnostic criteria. The genotyping results of candidate SNPs were obtained by the Sanger sequencing method. After completing the sequencing work for all the samples, the chi-square test on the gene frequency statistics was performed, and SNPs that showed significant differences in allele frequencies between the clinical ketosis group and the healthy group were identified (Figure 2). Our analysis revealed that four genes, namely *MAPK1* (chr17:74014223, T > G), *VEGFA* (chr23:17316741, C > T), *BoLA* (chr23:27671377, A > G), and *PTPRN2* (chr4:120051703, C > T), were all significantly associated with clinical ketosis in Chinese Holstein cows (*p* < 0.01). To further understand the nature of these associations, we calculated the odds ratios (OR) for each locus. In the clinical ketosis group, the *MAPK1* (rs74014223, T > G) locus had a significantly higher frequency of the alternative base G compared to the normal dairy group, with an OR of 2.925 (Figure 2B,F). Similarly, for the *VEGFA* (rs17316741, C > T) locus, the clinical ketosis group had a significantly greater proportion of the alternative base T, with an OR of 3.219 (Figure 2D,F). The *PTPRN2* (rs120051703, C > T) locus in the clinical ketosis group showed a highly significant increase in the frequency of the alternative base T, with an OR of 3.929 (Figure 2E,F). In contrast, the *BoLA* (rs27671377, A > G) locus presented a different pattern. The reference base A was more prevalent in the clinical ketosis group compared to the normal cow group, with an OR of 0.311 (Figure 2C,F). Since an OR value less than 1 indicates a protective factor, this suggests that the BoLA locus acts as a protective factor against clinical ketosis, while the other three loci (*MAPK1*, *VEGFA*, and *PTPRN2*) are risk factors for this disease. These results suggest that *MAPK1*, *VEGFA*, *PTPRN2,* and *BoLA* can serve as candidate genes for ketosis-related research and are suitable for subsequent in vivo and vitro validation. Among these genes, *MAPK1* (rs74014223, T > G) stood out as it had the smallest *p*-value in the genome-wide association analysis. Moreover, previous studies have reported that MAPK1 plays an important role in lipid metabolism. Given the close association between ketosis and lipid metabolism disorders in dairy cows, the present experiment will next focus on exploring whether MAPK1 is involved in lipid metabolism processes during the development of ketosis in dairy cows.

### 3.3. MAPK1 Plays an Important Role in Lipid Metabolism in Both Ketotic Cow Livers and Bovine Hepatocytes

**Figure 3:**
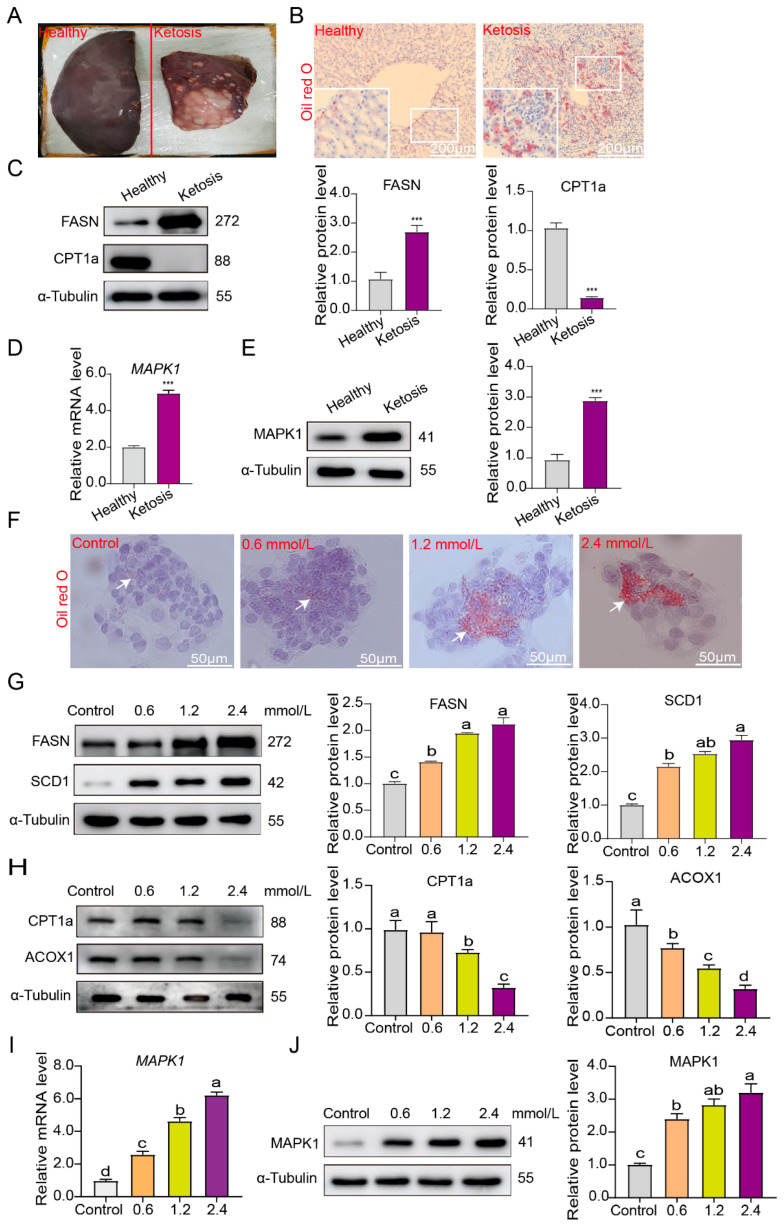
MAPK1 was significantly upregulated in liver tissue of the ketosis cows and in BHB-exposed bovine hepatocytes. (**A**) Liver tissue of healthy and ketosis cows. (**B**) Oil Red O staining analysis of liver tissues extracted from healthy and ketosis cows. Red indicates lipid-laden hepatocytes and white boxes indicates lipid droplets. (**C**) Western blot analysis of FASN and CPT1a protein levels using the samples extracted from cows’ healthy and ketotic liver tissues. (**D**) *MAPK1* gene expression level was detected in cows’ healthy and ketotic liver tissues via the qPCR method. (**E**) Western blot analysis of MAPK1 protein level using the samples extracted from cows’ healthy and ketotic liver tissues. (**F**) Oil Red O staining analysis of bovine hepatocytes stimulated by different concentrations of BHB: white arrows point to lipid droplets. (**G**,**H**) Western blot analysis of FASN, SCD1, CPT1a, and ACOX1 protein levels using the samples extracted from BHB-stimulated bovine hepatocytes. (**I**) *MAPK1* gene expression level was detected in BHB-stimulated bovine hepatocytes via the qRT-PCR method. (**J**) Western blot analysis of MAPK1 protein level using the samples extracted from BHB-stimulated bovine hepatocytes. The results are presented as Mean ± SD; *n* = 3. *** *p* < 0.001. Lowercase letters indicate significant level (*p* = 0.05).

In line with this objective, histological analysis was first carried out. The results revealed severe hepatic steatosis, disrupted lobular architecture, and significantly increased lipid accumulation in the livers of ketotic cows (Figure 3A,B). At the protein level, we observed that FASN expression was significantly elevated in ketotic cows, while CPT1a levels were markedly lower compared to those in healthy controls (Figure 3C). These findings clearly indicate that hepatocytes in ketotic cows are suffering from lipid metabolism dysfunction. Given that our previous studies have demonstrated that the *MAPK1* gene harbors major SNP associated with clinical ketosis, which implies its potential crucial role in the pathological process of this disease, we then examined the expression levels of MAPK1. As expected, we found that both its mRNA and protein levels were significantly upregulated in ketotic liver tissue when compared to healthy liver tissue (Figure 3D,E). To further validate the role of the candidate gene *MAPK1*, a lipid-metabolism-disturbed bovine hepatocyte model was established by treating with the concentrations of 0.6, 1.2, and 2.4 mmol/L β-hydroxybutyrate (BHB). Following Oil Red O staining, it was evident that the bovine hepatocytes exposed to 1.2 and 2.4 mmol/L β-hydroxybutyrate (BHB) exhibited a marked increase in intracellular lipid content (Figure 3F). We also observed distinct changes in the expression of lipid-metabolism-related proteins. The protein expression levels of FASN and SCD1, which are enzymes involved in fatty acid synthesis and desaturation, respectively, were significantly upregulated, while those of CPT1a and ACOX1, enzymes crucial for fatty acid oxidation, were markedly downregulated (Figure 3G,H), These protein expression patterns are consistent with the expected alterations in a cell model with disrupted lipid metabolism, thereby confirming the successful establishment of our in vitro lipid-metabolism-disturbed bovine hepatocyte model. Moreover, consistent with in vivo data, the mRNA and protein levels of MAPK1 rose accordingly. Specifically, MAPK1 expression was dose-dependently upregulated by BHB treatment (Figure 3I,J). Collectively, the combined experimental data from both in vivo and in vitro studies clearly demonstrate that MAPK1 expression is significantly elevated in both ketotic cow livers and bovine hepatocytes with lipid metabolism dysfunction.

### 3.4. MAPK1 Plays a Crucial Role in Lipid Metabolism Disorders in the Liver Caused by Ketosis in Dairy Cows

**Figure 4:**
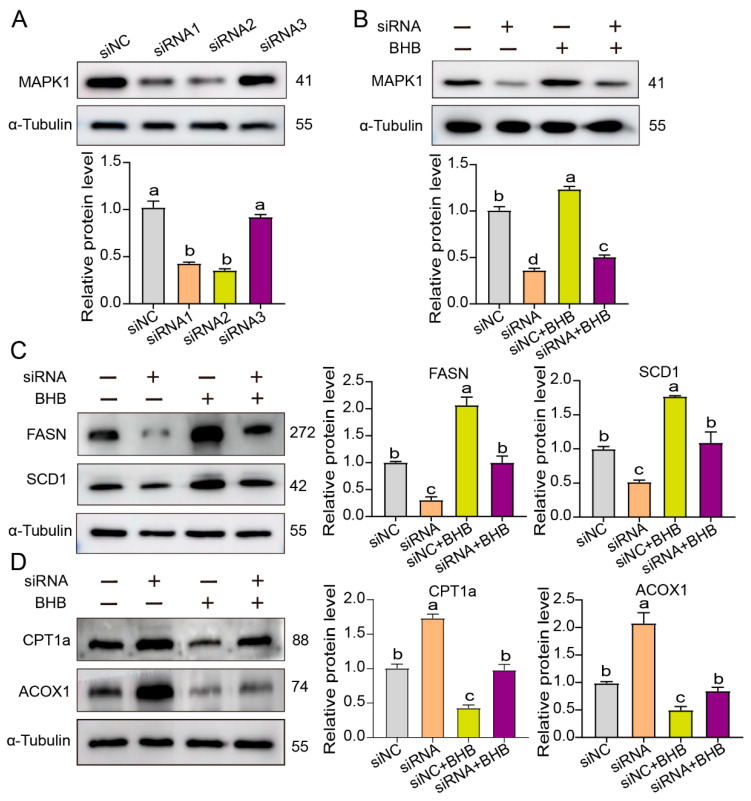
MAPK1 accelerated lipid accumulation and played a key role in lipid metabolism in BHB-exposed bovine hepatocytes. (**A**) Transfection efficiency of MAPK1–siRNA. (**B**) Western blot analysis of MAPK1 protein level using the samples extracted from MAPK1–siRNA and BHB-exposed bovine hepatocytes. (**C**,**D**) Western blot analysis of FASN, SCD1, CPT1a, and ACOX1 protein levels using the samples extracted from MAPK1–siRNA and BHB-exposed bovine hepatocytes. The results are presented as Mean ± SD; *n* = 3. Lowercase letters indicate significant level (*p* = 0.05).

To further investigate the potential involvement of MAPK1 in ketosis-related lipid metabolism disorders, MAPK1-knockdown bovine hepatocytes (siMAPK1) using RNA interference with siRNA were constructed. The results showed that the interference efficiencies of both siRNA1 and siRNA2 exceeded 80%, with siRNA2 showing a higher efficiency; thus, siRNA2 was selected for the subsequent experiments (Figure 4A). Following efficient knockdown of MAPK1 expression, we observed that the MAPK1 level was significantly downregulated in hepatocytes exposed to BHB compared to the non-knockdown control group under the same BHB treatment conditions (Figure 4B). As a result, FASN and SCD1 levels were significantly decreased in BHB-exposed hepatocytes (Figure 4C), while the expression levels of ACOX1 and CPT1a exhibited a significant upregulation when compared with the hepatocytes that had not undergone MAPK1 knockdown but were also exposed to BHB (Figure 4D). This opposite trend in the expression of these proteins upon MAPK1 knockdown under BHB-induced conditions further highlights the regulatory role of MAPK1 in lipid metabolism. Taken together, these findings suggest MAPK1 plays a critical role in regulating lipid metabolic dysfunction in bovine ketosis.

### 3.5. The FGF21–MAPK1 Signaling Pathway Participates in Lipid Metabolism Disorders in the Liver Caused by Ketosis in Dairy Cows

**Figure 5:**
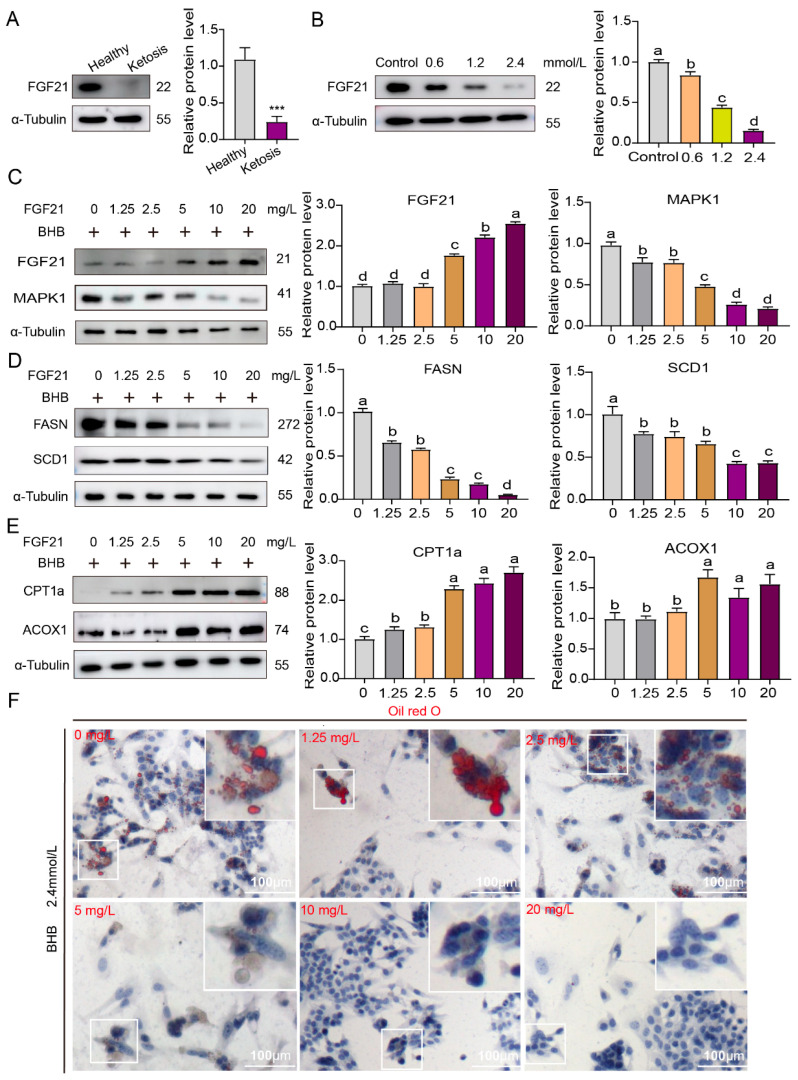
FGF21 involved the MAPK1 pathway and alleviated lipid accumulation in BHB-exposed bovine hepatocytes. (**A**) Western blot analysis of FGF21 protein levels using the samples extracted from cows’ healthy and ketotic liver tissues. (**B**) Western blot analysis of FGF21 protein level using the samples extracted from BHB-exposed bovine hepatocytes. (**C**–**E**) Western blot analysis of FGF21, MAPK1, FASN, SCD1, CPT1a, and ACOX1 protein levels using the samples extracted from bovine hepatocytes stimulated by different concentrations of FGF21 and 2.4 mmol/L BHB. (**F**) Oil Red O staining analysis of bovine hepatocytes stimulated by different concentrations of FGF21 and BHB: lipid droplets are in the white box. The results are presented as Mean ± SD; *n* = 3. *** *p* < 0.001. Lowercase letters indicate significant level (*p* = 0.05).

Given that MAPK1 has been identified as a crucial mediator through which FGF21 modulates lipid homeostasis and energy balance, and considering that FGF21 is a promising therapeutic target for addressing lipid metabolic dysfunction in obesity [21], our subsequent experiments specifically aimed to investigate whether FGF21 is involved in the MAPK1-mediated regulatory role in lipid metabolic disorders in the context of bovine ketosis. In our in vivo ketosis model, the results showed that the FGF21 expression level was notably lower than that in healthy controls (Figure 5A). Similarly, in the in vitro BHB-induced lipid-metabolism-disrupted hepatocyte model, the expression of FGF21 decreased in a concentration-dependent manner as the BHB levels increased (Figure 5B). These findings suggest a potential negative regulation effect of FGF21 on MAPK1 expression. To verify this hypothesis, we added an FGF21-Fc fusion protein to rescue FGF21 expression in the BHB-induced model. Consequently, MAPK1 expression was significantly downregulated (Figure 5C), a result similar to that of MAPK1 knockdown. This confirmed that FGF21 participates in regulating MAPK1 expression. To further substantiate FGF21′s role in mediating the effect of MAPK1 in ketosis, we analyzed whether rescuing FGF21 expression could impact lipid metabolism by assessing the expression levels of the aforementioned lipid-metabolism-related enzymes. Notably, FASN and SCD1 exhibited similar downregulation patterns similar to those in the MAPK1 knockdown group (Figure 5D). Conversely, CPT1a and ACOX1 were upregulated (Figure 5E). Furthermore, Oil Red O staining showed that the number of lipid droplets gradually decreased with increasing levels of FGF21-Fc fusion protein (Figure 5F). These findings demonstrate that rescuing FGF21 expression can effectively reverse the abnormal lipid-metabolism patterns induced by ketosis-related factors, likely through its interaction with MAPK1. This further confirms the significant role of FGF21 in mediating MAPK1′s effects on lipid metabolism during ketosis.

## 4. Discussion

Ketosis in dairy cows is a highly prevalent energy metabolism disorder that poses a serious threat to the health, reproductive capacity, and economic efficiency of dairy farms [3]. The incidence of the disease exhibits significant individual susceptibility differences, suggesting that genetic background plays a key role. Therefore, systematically screening for susceptibility candidate genes for ketosis in dairy cows is of critical importance—this not only reveals the molecular genetic mechanisms underlying the disease but also serves as a prerequisite for achieving early risk warning and precise prevention and control. In this study, the key finding emerging from this study is that four SNPs (*MAPK1*, *BoLA, VEGFA*, and *PTPRN2*) are associated with clinical ketosis in Chinese Holstein cows. Ketosis in dairy cattle is intricately linked to disruptions in glucose and lipid metabolism. Bovine leucocyteantigen (*BoLA*) genes, also known as major histocompatibility complex (MHC) genes, are a family of genes consisting of compactly linked polymorphic loci. It has been shown that bovine MHC gene polymorphisms are associated with resistance and susceptibility to a variety of diseases, such as mastitis [22] and foot-and-mouth disease [23]. Population validation of the *BoLA* (rs27671377, A > G) polymorphism revealed it to be a protective factor against ketosis (OR < 1), indicating its potential role in conferring resistance to this metabolic disorder. Separately, studies on the protein tyrosine phosphatase receptor N2 (PTPRN2, also known as phogrin or IA-2β) knockout murine models demonstrated mild glucose intolerance [24] and modest reductions in pancreatic insulin content [25]. While PTPRN2 has been extensively characterized in the context of insulin resistance, its involvement in lipid metabolism remains sparsely explored. Our data uniquely demonstrate that PTPRNT2 may play an important role in hepatic lipid metabolism. Vascular endothelial growth factor A (VEGFA) is a critical mediator of angiogenesis, vasculogenesis, vascular permeability, and tissue remodeling [26]. However, VEGFA also exhibits significant metabolic functions, modulating systemic energy metabolism through both its upregulation and downregulation [27]. Notably, adipose tissue-specific overexpression of VEGFA confers protection against diet-induced obesity and insulin resistance in murine models [28]. Given the established association between ketone bodies and insulin resistance disorders [29], it is plausible that genes implicated in insulin resistance pathways, including PTPRN2 and VEGFA, may also contribute to the underlying metabolic dysregulation of ketosis since MAPK1 had the smallest *p* value in the genome-wide association analysis. Next, we will further detect whether and how MAPK1 affects hepatic lipid metabolism in ketosis cows.

Ketosis represents a major contributor to diminished lactation performance in dairy cows, particularly high-yielding lactating individuals. The pathogenesis of this metabolic disorder involves dysregulation of fatty acid metabolism, encompassing uptake, transport, activation, oxidation, synthesis, and esterification [30]. MAPK1, a core component of the MAPK signaling pathway, not only regulates cell proliferation and differentiation but also modulates fatty acid metabolism through multiple mechanisms. Therefore, after identifying candidate genes through techniques such as GWAS, rigorous biological functional validation is indispensable. We have identified *MAPK1* was significantly elevated in an in vivo and in vitro model of ketosis. Hepatic activation of MAPK1 inhibits the transcriptional activity of PPARγ via phosphorylation at Ser112, consequently downregulating key lipogenic enzymes such as acetyl-CoA carboxylase (ACC) and fatty acid synthase (FASN) [31]. Furthermore, catecholamine-induced MAPK1 activation stimulates lipolysis, elevating circulating free fatty acid (FFA) levels—a process implicated in the hepatic lipid accumulation characteristic of bovine ketosis [32]. This explains the aggregation of lipid droplets in the liver tissue of cows with ketosis. Evidence also indicates that MAPK1 activation promotes peripheral insulin resistance and enhances pancreatic β-cell apoptosis [33]. Under insulin-resistant conditions, aberrant MAPK1 signaling may impair fatty acid β-oxidation by suppressing the AMPK pathway. Notably, ketone bodies themselves contribute to insulin resistance by downregulating cell surface insulin receptors and inhibiting insulin receptor substrate-1 (IRS-1) phosphorylation [34]. These observations provide a mechanistic rationale for the significant alterations in MAPK1 signaling observed following exposure to β-hydroxybutyrate (BHB), the predominant ketone body.

In order to explore how MAPK1 plays a role in hepatic lipid metabolism in ketosis cows, we performed MAPK1 knockdown experiments in an in vitro model of ketosis. As previously reported, MAPK1 serves as a critical regulator of lipid metabolism across multiple tissue types. In colorectal carcinogenesis, MAPK1 promotes lipid metabolic reprogramming [35]. Studies on lung cancer cells have shown that MAPK1 phosphorylation inhibits FASN expression [15], while MAPK1 activation enhances the expression of fatty acid metabolism genes such as CPT1a in diabetic cardiomyopathy [36]. These collective findings establish MAPK1 as a central node in lipid homeostasis. So, when MAPK1 is inhibited, can lipid metabolism, particularly in the liver, be reversed? Complementary evidence from nutritional interventions reveals that purple sweet potato color (PSPC) attenuates MAPK signaling in murine liver, concomitantly reducing lipogenic (FASN) and fatty acid uptake (CD36) markers while elevating β-oxidation proteins (CPT1a) [37]. Pharmacological inhibition studies further corroborate MAPK1′s role in ameliorating saturated fatty acid (SFA)-induced hepatic lipid accumulation and cellular demise [38]. From the above discussion, the conclusion can be reached that MAPK1 plays an important regulatory role in lipid metabolism. Our current findings align with those of older studies: MAPK1 knockdown significantly upregulated β-oxidation factors while downregulating lipogenic factors in a ketosis model. Collectively, these data implicate MAPK1-mediated dysregulation of hepatic lipid metabolism as a fundamental mechanism underlying ketosis pathogenesis in dairy cattle.

Our above findings confirm that MAPK1 plays a key role in the development of ketosis in dairy cows, so is there a potential target that can act on MAPK1 to alleviate the symptoms of ketosis in dairy cows? We found that fibroblast growth factor 21 (FGF21) plays a crucial role in regulating glucose homeostasis, lipid metabolism, and insulin sensitivity [17]. Our experimental results reflect that FGF21 has an important role in reducing hepatic lipid accumulation in a ketosis model. The FGF21–MAPK1 signaling axis, extensively characterized in metabolic tissues such as liver and muscle [39,40], represents a key regulatory pathway. In dairy cattle, FGF21 has emerged as a promising biomarker for ketosis detection [16] and is implicated in early lactation adaptations. Furthermore, FGF21 is widely investigated as a therapeutic target for lipid-lowering interventions [21]. Consistent with prior reports [18], our data demonstrate that BHB stimulation significantly suppresses FGF21 expression, revealing a significant negative correlation with MAPK1 levels. Mechanistically, FGF21 modulates central lipid metabolic enzymes—downregulating fatty acid synthase while upregulating CPT1a via diverse signaling pathways [41,42,43]. This aligns with clinical evidence, wherein FGF21 infusion reduced hepatic triglyceride content by 50% in liver biopsies [44]. Critically, our experiments in BHB-exposed bovine hepatocytes show that elevating FGF21 concentrations significantly decreased expression of lipogenic genes (FASN, SCD1) and concurrently increased expression of β-oxidation genes (CPT1a, ACOX1). These findings position FGF21 as a critical mediator of energy metabolism adaptation and MAPK1 pathway dysregulation during ketosis pathogenesis. While our work establishes FGF21′s role in hepatic lipid reprogramming, the precise mechanisms by which the FGF21–MAPK1 axis governs lipid-metabolizing gene expression warrant further investigation. Collectively, these results provide foundational insights supporting FGF21 as a potential therapeutic target for ketosis in lactating dairy cattle.

## 5. Conclusions

**Figure 6:**
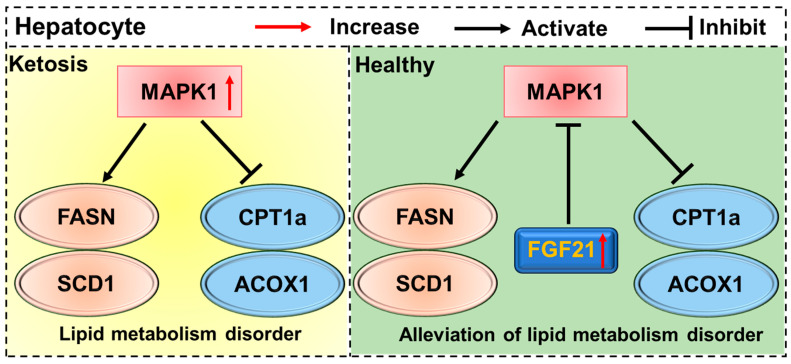
The FGF21–MAPK1 signaling axis exists in hepatocytes with lipid metabolism disorder.

In conclusion, this study systematically screened candidate genes for bovine ketosis and identified four significant SNPs out of seventeen SNPs associated with clinical ketosis. Among them, the MAPK1 variant exhibited the strongest association. Further research demonstrated that MAPK1 upregulation disrupts hepatic lipid homeostasis in dairy cow ketosis. Notably, the downregulation of FGF21 in ketosis models implies that supplementing FGF21 could reduce MAPK1 expression and normalize lipid metabolism (Figure 6). Additionally, studies involving larger sample sizes and breed-specific groups would be valuable in confirming and extending these results. Future research should expand on these findings by examining breed-specific variations in bovine ketosis. These findings establish the FGF21–MAPK1 imbalance as a key cause of hepatic lipid metabolic dysfunction, offering a potential intervention strategy for dairy cow ketosis.

## Figures and Tables

**Table 1:** Candidate genes significantly associated with ketosis and their main functions.

CHR	POS	Gene	Function	Reference
1	95,777,224	*GHSR*	Insulin secretion or glucose tolerance	Gray SM et al., 2019
1	120,339,915	*AGTR1*	Insulin secretion	Shoemaker R et al., 2019
			Promotes hepatic mRNA expression of cholesterol–metabolism-related genes	Pellegrin M et al., 2018
4	120,051,703	*PTPRN2*	Inhibit glucose-stimulated insulin secretion	Wang Y et al., 2010
			Insulin signaling	Torii S et al., 2018
7	97,574,524	*GLRX*	A new target for diabetes mellitus Type 2 treatment	Petry SF et al., 2017
11	9,371,465	*TACR1*	Glucose metabolism	Karagiannides I et al., 2011
13	19,864,372	*NRP1*	Weight gain and glucose tolerance	Wilson AM et al., 2018
15	4,715,091	*PDGFD*	Promotes cell proliferation, cell migration, and expression of inflammatory factors in adventitial fibroblasts	Zhang ZB et al., 2018
15	32,076,300	*GRIK4*	Glutamatergic synapse	Catches JS et al., 2012
17	74,014,223	*MAPK1*	Insulin secretion	Niu B et al., 2016
19	54,453,831	*SOCS3*	Mediates insulin and leptin	Yang Z et al., 2012
23	7,799,625	*IP6K3*	Ip6k3(-/-) mice had lower blood glucose, less insulin, decreased fat, lower weight	Yusuke M et al., 2016
23	17,316,741	*VEGFA*	Regulates adipose development and function in energy metabolism	Jin H et al., 2018
23	27,671,377	*BoLA*	Type 1 diabetes mellitus	Finer S et al., 2016
24	48,201,784	*ZBTB7C*	A crucial metabolic regulator of blood glucose homeostasis	Won-II C et al., 2019
29	22,755,876	*SLC17A6*	Vglut2 conditional knockout mice showed less interest in fatty rewards and decreased sugar consumption	Schweizer N et al., 2016
29	41,070,494	*FADS2*	Hepatic lipid accumulation; secretion of very low-density lipoprotein (VLDL)	Hayashi Y et al., 2018
30	62,655,807	*ACSL4*	Adipose tissue inflammation and insulin resistance	Elizabeth A K et al., 2018

CHR, chromosome; POS, position.

**Table 2:** Loci that are significantly associated with ketosis are associated with glucose and lipid metabolism.

Locus Name	SNP	A1/A2	Freq.A1	He	Ho	PIC	BETA	*p* Value
*LOC107133317-**MAPK1***	rs74014223	T/G	0.913	0.1597	0.175	0.1469	0.181	3.74 × 10^−5^
*TNFSF10-**GHSR***	rs95777224	A/G	0.946	0.1023	0.1081	0.097	0.236	1.17 × 10^−5^
***IP6K3***	rs7799625	T/C	0.054	0.1023	0.1081	0.097	0.212	0.000148
***SLC17A6*** *-ANO5*	rs22755876	C/T	0.060	0.112	0.119	0.1057	0.211	0.000186
***PDGFD***	rs4715091	G/T	0.941	0.112	0.119	0.1057	0.188	0.000197
*PGS1-**SOCS3***	rs54453831	A/C	0.917	0.1528	0.1667	0.1411	0.157	0.00021
***PTPRN2***	rs120051703	C/T	0.769	0.355	0.4103	0.292	0.113	0.000218
***BoLA***	rs27671377	A/G	0.597	0.4811	0.6944	0.3654	0.118	0.000266
***FADS2***	rs41070494	G/A	0.842	0.2668	0.3171	0.2312	0.132	0.000459
***GRIK4***	rs32076300	G/A	0.872	0.2235	0.2051	0.1986	0.121	0.000523
***ZBTB7C***	rs48201784	C/G	0.081	0.149	0.1622	0.1379	0.162	0.000664
*CPB1-**AGTR1***	rs120339915	G/A	0.915	0.1562	0.122	0.144	0.128	0.000692
***GLRX*** *-ELL2*	rs97574524	G/A	0.907	0.1687	0.186	0.1545	0.146	0.000701
***VEGFA*** *-C23H6orf223*	rs17316741	C/T	0.865	0.2337	0.2162	0.2064	0.119	0.000816
***ACSL4*** *-LOC781152*	rs62655807	T/C	0.927	0.1356	0.1463	0.1264	0.143	0.000876
*PARD3-**NRP1***	rs19864372	A/T	0.919	0.1495	0.1628	0.1384	0.145	0.000951
*FHL2-**TACR1***	rs9371465	G/A	0.540	0.4969	0.5526	0.3734	0.089	0.000998

Note: A1, reference base; A2, substitute base; He, expected heterozygosity; Ho, observed heterozygosity; PIC, polymorphism information content. Bolded are the candidate genes.

## Data Availability

The data presented in the study are deposited in the Genome Sequence Archive repository (https://ngdc.cncb.ac.cn/gsa/), accession number CRA013801, accessed on 8 December 2023.

## Supplementary Materials

The following supporting information can be downloaded at: https://www.mdpi.com/article/10.3390/life15091339/s1, Table S1: DNA quality test results of whole blood of 45 sequenced cows; Table S2: Primer information for Sanger sequencing PCR; Table S3: qRT-PCR Primer sequences; Table S4: MAPK1-siRNA Primer sequences.
