# Supplementary material for: FGF21–MAPK1 Imbalance Disrupts Hepatic Lipid Metabolism in Dairy Cow Ketosis
Source: Life (Basel). 2025 Aug 24;15(9):1339. doi: 10.3390/life15091339 (PMC12470934; doi:10.3390/life15091339)
Supplement: Supplementary file 1 [file life-15-01339-s001.zip › Figure S1.pdf]

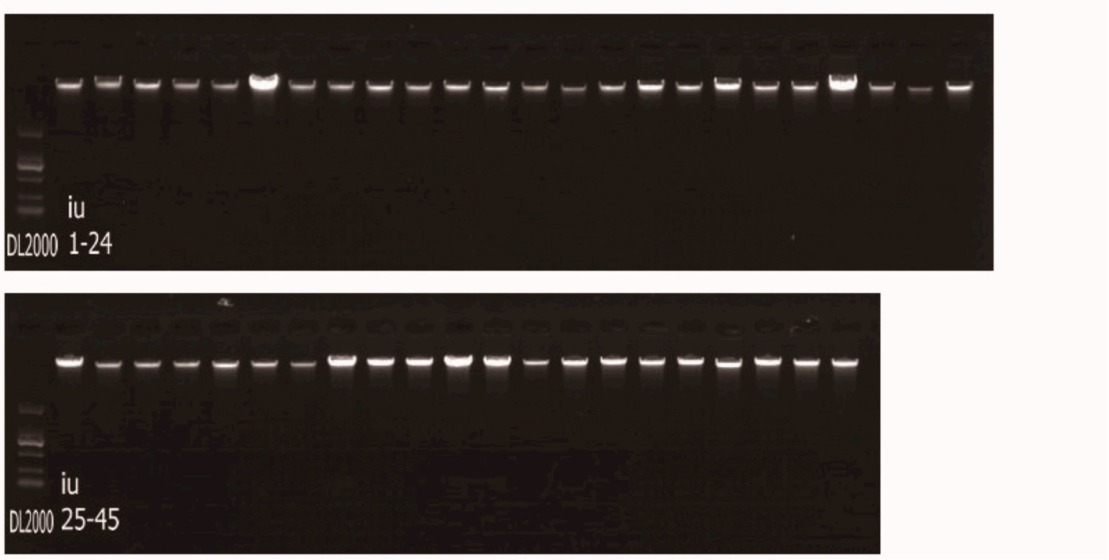

Figure S1. Agarose gel electrophoresis of whole blood DNA of 45 sequenced cows. iu 1-45: sequencing sample number; DL2000: DNA Marker.
