# Supplementary material for: FGF21–MAPK1 Imbalance Disrupts Hepatic Lipid Metabolism in Dairy Cow Ketosis
Source: Life (Basel). 2025 Aug 24;15(9):1339. doi: 10.3390/life15091339 (PMC12470934; doi:10.3390/life15091339)
Supplement: Supplementary file 1 [file life-15-01339-s001.zip › Supplementry TableS1-S4.pdf]

**Table S1**

Table S1 DNA quality test results of whole blood of 45 sequenced cows

| Sequencing number | Sample number | Postpartum milk BHB（mmol/L） | Concentration（ng/μL） | Volume（μL） | A260/280 | A260/230 | Quality analysis |
|---|---|---|---|---|---|---|---|
| iu-1 | K1 | 0.22 | 30.7 | 30 | 1.88 | 1.55 | A |
| iu-2 | K2 | 0.21 | 44.1 | 30 | 1.91 | 1.84 | A |
| iu-3 | K3 | 0.21 | 48.8 | 30 | 1.89 | 1.85 | A |
| iu-4 | K4 | 0.44 | 39 | 30 | 1.88 | 1.56 | A |
| iu-5 | K5 | 0.21 | 39.1 | 30 | 1.9 | 1.63 | A |
| iu-6 | K6 | 0.54 | 152.3 | 30 | 1.87 | 2.11 | A |
| iu-7 | K7 | 0.2 | 37.4 | 30 | 1.88 | 1.58 | A |
| iu-8 | K8 | 0.29 | 36.8 | 30 | 1.8 | 1.5 | A |
| iu-9 | K9 | 0.28 | 30 | 30 | 1.94 | 1.5 | A |
| iu-10 | K10 | 0.20 | 31.6 | 30 | 1.89 | 1.37 | A |
| iu-11 | K11 | 0.41 | 47.1 | 30 | 1.86 | 1.58 | A |
| iu-12 | K12 | 0.21 | 36.5 | 30 | 1.91 | 1.56 | A |
| iu-13 | K13 | 0.29 | 30.7 | 30 | 1.91 | 1.64 | A |
| iu-14 | K14 | 0.31 | 15.1 | 30 | 1.95 | 1.39 | A |
| iu-15 | K15 | 0.28 | 38.8 | 30 | 1.89 | 1.75 | A |
| iu-16 | K16 | 0.46 | 28.7 | 30 | 1.92 | 1.76 | A |
| iu-17 | K17 | 0.24 | 34.5 | 30 | 1.87 | 1.59 | A |
| iu-18 | K18 | 0.20 | 49.4 | 30 | 1.86 | 1.74 | A |
| iu-19 | K19 | 0.23 | 25.3 | 30 | 1.9 | 1.5 | A |
| iu-20 | K20 | 0.22 | 26.5 | 30 | 1.89 | 1.58 | A |
| iu-21 | K21 | 0.22 | 109.7 | 30 | 1.87 | 1.99 | A |
| iu-22 | K22 | 0.27 | 17.8 | 30 | 1.97 | 1.57 | A |
| iu-23 | K23 | 0.32 | 14.5 | 30 | 1.88 | 1.13 | A |
| iu-24 | K24 | 0.24 | 26.5 | 30 | 1.86 | 1.42 | A |
| iu-25 | K25 | 0.21 | 58.7 | 30 | 1.86 | 1.73 | A |
| iu-26 | C1 | 0.13 | 33 | 30 | 1.95 | 1.5 | A |
| iu-27 | C2 | 0.06 | 31.1 | 30 | 1.91 | 1.49 | A |
| iu-28 | C3 | 0.07 | 20.7 | 30 | 1.91 | 1.33 | A |
| iu-29 | C4 | 0.11 | 27.3 | 30 | 1.86 | 1.52 | A |
| iu-30 | C5 | 0.1 | 30.4 | 30 | 1.87 | 1.46 | A |

| | | | | | | | |
|---|---|---|---|---|---|---|---|
| iu-31 | C6 | 0.09 | 18 | 30 | 1.78 | 1.19 | A |
| iu-32 | C7 | 0.12 | 80.2 | 30 | 1.87 | 1.97 | A |
| iu-33 | C8 | 0.12 | 56.2 | 30 | 1.88 | 1.77 | A |
| iu-34 | C9 | 0.10 | 59.8 | 30 | 1.84 | 1.23 | A |
| iu-35 | C10 | 0.14 | 90.9 | 30 | 1.85 | 1.74 | A |
| iu-36 | C11 | 0.09 | 63.4 | 30 | 1.87 | 1.94 | A |
| iu-37 | C12 | 0.11 | 25.2 | 30 | 1.91 | 1.22 | A |
| iu-38 | C13 | 0.13 | 43.4 | 30 | 1.92 | 1.63 | A |
| iu-39 | C14 | 0.14 | 42.2 | 30 | 1.91 | 1.82 | A |
| iu-40 | C15 | 0.13 | 41.3 | 30 | 1.88 | 1.55 | A |
| iu-41 | C16 | 0.12 | 41.8 | 30 | 1.88 | 1.78 | A |
| iu-42 | C17 | 0.09 | 48.8 | 30 | 1.86 | 1.68 | A |
| iu-43 | C18 | 0.1 | 48.5 | 30 | 1.86 | 1.79 | A |
| iu-44 | C19 | 0.13 | 27.4 | 30 | 1.9 | 1.84 | A |
| iu-45 | C20 | 0.1 | 46.5 | 30 | 1.86 | 1.7 | A |

Note: K represents clinical ketosis group, C represents normal control group; A means that the quality meets the experimental requirements, and subsequent experiments can be carried out

## Table S2

Table S2 Primer information for Sanger sequencing PCR

| SNP | Genes | Primer sequencing | Annealing Temperature | Product size |
|---|---|---|---|---|
| AC_000174.1: 74014223 (T/G) | *LOC107133317-MAPK1*$^{\Delta}$ | F: TCGGGTTTGAGGAGTGCTTT<br>R: CATGGAAGCCAGGTAACTCTCA | 57 | 562 |
| AC_000172.1: 4715091 (G/T) | *PDGFD* | F: GAGAATACTGTTGGCGCCTG<br>R: ATGGACGGAGGAGCCTGTTA | 57 | 346 |
| AC_000186.1: 22755876 (C/T) | *SLC17A6*$^{\Delta}$*-ANO5* | F: TCTGATGCTGGGAGGGATTG<br>R: AGCTGAAGCAGTACACTCACC | 62.2 | 565 |
| AC_000176.1: 54453831 (A/C) | *PGS1-SOCS3*$^{\Delta}$ | F: GGGAAGTCAGTGGCTCAAAGT<br>R: AGGAATAAACAGCCCCTGAGC | 57 | 330 |
| AC_000180.1: 27671377 (A/G) | *BoLA* | F: TGCCCATGTGACCATTACCAC<br>R: CCCACAAAGAATTACAGCCAGC | 57 | 412 |
| AC_000172.1: 32076300 (G/A) | *GRIK4* | F: TGAAGCCACATGGGTACGAT<br>R: GGCACACAGGCTTCTTAGACT | 57 | 411 |
| AC_000158.1: 120339915 (G/A) | *CPB1-AGTR1*$^{\Delta}$ | F: AGAGTTCACACGCTGCAACTA<br>R: TTGTGTTCGACTCTGTGCGA | 63 | 420 |
| AC_000187.1: 62655807 (T/C) | *ACSL4*$^{\Delta}$*-LOC781152* | F: AACACCCAGGACTGTTCTCC<br>R: ATGACACCACCCTTATGGCAG | 57.5 | 493 |

| | | | | |
|---|---|---|---|---|
| AC_000180.1:<br>17316741<br>（C/T） | *VEGFA*$^{\Delta}$*-C23H6orf223* | F: AGTGCGTCAACACTGGATTG<br>R: CCCAGGTCAATGACTTCTGGA | 57 | 421 |
| AC_000168.1:<br>9371465<br>（G/A） | *FHL2-TACR1*$^{\Delta}$ | F: GGGAAATGGTGGAAACAGTGG<br>R: TCAGGCCTTCCAATGAACACC | 63 | 364 |
| AC_000158.1:<br>95777224<br>（A/G） | *TNFSF10-GHSR*$^{\Delta}$ | F: TATTTGTGGGCTTCCCCGGT<br>R: GGAATGAACTGATGGCGGAC | 57 | 355 |
| AC_000180.1:<br>7799625<br>（T/C） | *IP6K3* | F: CCATTGCCTTCTCCACAACG<br>R: TGGCTAGGGCAGAAAGTTCC | 55.8 | 632 |
| AC_000161.1:<br>120051703<br>（C/T） | *PTPRN2* | F: ATTGTAGCATGTCGCGTTGG<br>R: GCATGACTATGGGCGCATTC | 60.4 | 564 |
| AC_000186.1:<br>41070494<br>（G/A） | *FADS2* | F: TGACAGGCGATCTTGCAGTC<br>R: GAAACAGGCCCCAACTTTGC | 59.4 | 685 |
| AC_000181.1:<br>48201784<br>（C/G） | *ZBTB7C* | F: TTCCAAGTGACTCTCCCGTG<br>R: GCAATGAACCAGGTATTGGGC | 56.6 | 780 |
| AC_000164.1:<br>97574524<br>（G/A） | *GLRX*$^{\Delta}$*-ELL2* | F: GGCAGAGCTTTTACCGACTG<br>R: GCCACCTCTGTCATGAAACC | 61.4 | 610 |
| AC_000170.1:<br>19864372<br>（A/T） | *PARD3-NRP1*$^{\Delta}$ | F: GAGACACGTGCTGACAAAGC<br>R: AGTTGCACCCCAAGATCACC | 55 | 621 |

## Table S3

Table S3 qRT-PCR Primer sequences

| Gene | | Primer sequences |
|---|---|---|
| *MAPK1* | sense | 5’-GTCGCCATCAAGAAAATCAGC-3’ |
| | antisense | 5’-GGAAGGTTTGAGGTCACGGT-3’ |
| *β-Actin* | sense | 5’-GGGCAGGTCATCACCATCGG-3’ |
| | antisense | 5’-TCATTGTGCTGGGTGCCAGG-3’ |

## Table S4

TableS4 MAPK1-siRNA Primer sequences

| Gene | | Primer sequences |
|---|---|---|
| *siMAPK1-1* | sense | 5’-GCAAGUACAUCAAGGUCAU-3’ |
| | antisense | 5’-AUGACCUUGAUGUACUUGC-3’ |
| *siMAPK1-2* | sense | 5’-CCUGGAGUUCAUCAAGAAU-3’ |
| | antisense | 5’-AUUCUUGAUGAACUCCAGG-3’ |
| *siMAPK1-3* | sense | 5’-GGACAUCAUCUACGAGUUA-3’ |
| | antisense | 5’-UAACUCGUAGAUGAUGUCC-3’ |
| *siNegative control* | sense | 5’-UUCUCCGAACGUGUCACGU-3’ |
| | antisense | 5’-ACGUGACACGUUCGGAGAA-3’ |
